# Minimally invasive magnetic resonance image-guided prostate interventions

**DOI:** 10.1259/bjr.20210698

**Published:** 2021-11-01

**Authors:** Annemarijke van Luijtelaar, Jurgen J Fütterer, Joyce GR Bomers

**Affiliations:** 1 Department of Radiology and Nuclear Medicine, Radboud University Medical Center, Nijmegen, The Netherlands

## Abstract

Whole gland prostate cancer treatment, *i.e*. radical prostatectomy or radiation therapy, is highly effective but also comes with a significant impact on quality of life and possible overtreatment in males with low to intermediate risk disease. Minimal-invasive treatment strategies are emerging techniques. Different sources of energy are used to aim for targeted treatment in order to reduce treatment-related complications and morbidity. Imaging plays an important role in targeting and monitoring of treatment approaches preserving parts of the prostatic tissue. Multiparametric magnetic resonance imaging (mpMRI) is widely used during image-guided interventions due to the multiplanar and real-time anatomical imaging while providing an improved treatment accuracy. This review evaluates the available image-guided prostate cancer treatment options using MRI or magnetic resonance imaging/transrectal ultrasound (MRI/TRUS)-fusion guided imaging. The discussed minimal invasive image-guided prostate interventions may be considered as safe and feasible partial gland ablation in patients with (recurrent) prostate cancer. However, most studies focusing on minimally invasive prostate cancer treatments only report early stages of research and subsequent high-level evidence is still needed. Ensuring a safe and appropriate utilization in patients that will benefit the most, and applied by physicians with relevant training, has become the main challenge in minimally invasive prostate cancer treatments.

## Introduction

Traditionally, definitive treatment of a prostate tumor includes whole gland therapy, regardless the size or location of the tumor. Whole gland prostate cancer treatments includes radical prostatectomy (RP) or radiation therapy (RT) and is used for treating intermediate- to high-risk prostate cancer. Patients with low-risk prostate cancer mostly opt for an active surveillance policy.^1^ Despite the radical nature of these elaborate approaches, 40% of the patients undergoing definitive therapy will develop a biochemical recurrence.^2,3^ Furthermore, whole gland treatment also comes with treatment-related complications and side-effects which results in decreased functional outcome measurements.^4,5^

In the last decade, several minimal-invasive treatment strategies are emerging. These treatment methods aim for local destruction of cancerous cells using various sources of energy.^6^ The main advantage of these techniques is that preservation of the adjacent structures more likely because of the minimal-invasive approach and consequently treatment-related complications and morbidity could be reduced.^7^ Recent studies demonstrate that post-treatment prognosis is predominantly driven by the largest lesion with the highest grade, the so-called “index lesion”.^7,8^ Treatment approaches which preserve parts of the prostatic gland are considered as partial gland ablation and include “hockey stick” ablation, hemi-ablation, and focal ablation. Consequently, imaging plays an important role in detection, localization, targeting and monitoring partial gland prostate cancer treatments.

Multiparametric magnetic resonance imaging (mpMRI) is preferred in detecting and staging prostate cancer due to excellent soft tissue contrast and multiplanar anatomical imaging.^9,10^ It is also used to differentiate between post-treatment changes and potential recurrent or residual disease.^11^ As such, secondary treatment can be promptly established.^12^ More recently, mpMRI has gained acceptance in image-guided therapeutic settings since it offers real-time anatomical imaging in different planes and therefore improved treatment accuracy.^13^ Furthermore, it can provide real-time temperature imaging. However, using MRI during prostate cancer treatment can be time-consuming, expensive and the availability is limited to centers with experienced (interventional) radiologists and urologists. Therefore, magnetic resonance imaging/transrectal ultrasound (MRI/TRUS)-fusion guided treatments in which previously obtained mpMRI is cognitively or software-assisted fused with real-time TRUS images are more and more performed. Using MRI/TRUS fusion has the benefit of relatively low-costs because no MRI-compatible equipment and extra MR-scanner time are needed and it is readily available compared to the use of MRI. However, accurate alignment of the MR images is essential for a successful image registration and some MR reading experience is needed for accurate interpretation of the images.

The aim of this review is to provide an overview of the available techniques for image-guided prostate interventions using MRI and MRI/TRUS-fusion imaging. Table 1 presents an overview of the described image-guided intervention methods.

**Table 1.** An overview of the different image guided prostate interventions

Intervention method	Treatment principle	Mechanism	Imaging modality	Administration	Application
*Laser therapy*	Thermal	Energy provided by the laser fibers raises the temperature of the targeted tissue above 60°C, this results in direct focused cell death.	MRI-, TRUS- or MRI/TRUS-fusion guidance	Transrectal or transperineal	Focal Primary, salvage
*Cryoablation*	Thermal	Alternating freeze and thaw cycles induce cell death by cellular dehydration due to the decreased temperature (−40°C) of extracellular water and results in an osmotic gradient followed by coagulative necrosis, thrombosis and tissue ischemia.	MRI-, TRUS- or MRI/TRUS-fusion guidance	Transperineal	Focal, whole gland Primary, salvage
*High-intensity focused ultrasound*	Thermal	Energy from a high-frequency ultrasound is used to heat (>60°C) the targeted tissue and subsequently induces immediate and irreversible tumor necrosis and cause cavitation with sharply delineated margins.	MRI-, TRUS- or MRI/TRUS-fusion guidance	Transperineal	Focal Primary, Salvage
*Transurethral ultrasound ablation*	Thermal	The urethral applicator provides a beam of focused energy in order to achieve a temperature of >55°C which induces thermal coagulation of the prostatic tissue.	MRI-guidance	Transurethral	Focal, whole gland Primary, salvage
*Photodynamic therapy*	Reactive oxygen species	Energy transfer from the activated photosensitizing drug to biological substrates or molecular oxygen, generates reactive oxygen species which will induce cell death by apoptosis or necrosis	TRUS or MRI/TRUS-fusion guidance	Intravenous administration of photosensitizing drug and transperineal inserted laser fibers.	Focal, whole gland Primary, salvage
*Radiofrequency ablation*	Thermal	Delivery of low-dose radiofrequency waves directly to the targeted tissue induces irreversibly damage by coagulative necrosis and atrophy	TRUS or MRI/TRUS- fusion guidance	Transperineal	Focal. Primary, salvage
*Irreversible electroporation*	Electrical currents	Electrical pulses produce irreversible cell membrane permeabilization which causes apoptosis of the cells.	TRUS or MRI/TRUS–fusion guidance	Transperineal	Focal Primary, salvage
*Brachytherapy*	Radiation	Radioactive seeds that are implanted within the prostatic tissue	MRI-, TRUS- or MRI/TRUS-fusion guidance	Transperineal	Focal, whole gland Primary, salvage
*Radiation therapy*	Radiation	Focal stereotactic radiation therapy	MRI-guidance	n/a	Focal, whole gland Primary, salvage

MRI, Magnetic Resonance Imaging; TRUS, Transrectal ultrasound.

### Laser therapy

Prostate cancer treatment using focal laser ablation (FLA) or Laser Interstitial Thermo Therapy (LITT) is based on thermal ablation. An optical laser fiber is applied within the cancerous tissue by either a transrectal or transperineal approach. Energy provided by the laser raises the temperature of the targeted tissue above 60°C, this results in direct focused cell death. The number of ablations that is needed to cover the targeted area depends on the volume of the intended lesion and the type of laser fiber that is used. The total procedure time depends on the number of ablations that is needed, with an average of only a few minutes per fiber position. This results in short procedure times and a fast post-treatment recovery. Most importantly, secondary interventions (*i.e.* repeat FLA or radical treatment) remain a viable option after initial treatment with FLA.

The treatment can be performed under either MRI-, TRUS- or MRI/TRUS-fusion guidance. When performing the FLA procedure under MRI guidance, real-time MRI temperature mapping can be used. This allows real-time feedback of the heat distribution within the prostatic tissue and contributes to a constrained impact on adjacent structures which results in preserving urinary and sexual function. During TRUS- and MRI/TRUS fusion-guided treatments, temperature sensors can be used to measure the temperature near critical structures. Consequently, FLA has become a targeted and predictable, minimally invasive prostate cancer intervention that can be used in carefully selected patients.

**Figure 1.**
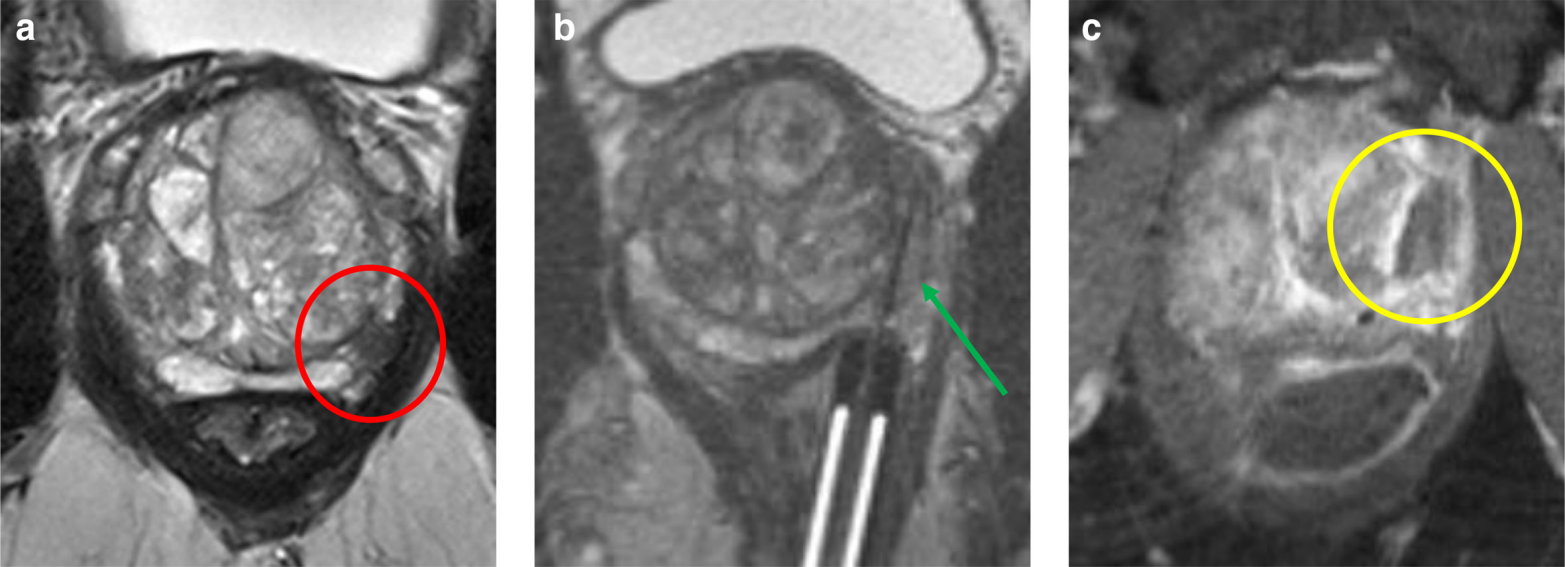
Transrectal focal laser ablation MRI of a 69-year-old male with an initial PSA of 14.5 ng ml^−1^ that underwent transrectal FLA as treatment for a *de novo* lesion (Gleason score 3+4=7) at the left peripheral zone (PI-RADS 4). (a) Axial *T*_2_W imaging of the apex with the prostate tumor (red circle) at the left peripheral zone; (b) Intraprocedural axial *T*_2_W imaging of the apex with the laser fiber (green arrow) *in situ*; (c) Axial *T*_1_W directly after treatment shows the ablation zone (yellow circle). FLA, Focal laser ablation; PI RADS, Prostate imaging- reporting and data system; PSA, Prostate specific antigen; *T*_1_W, *T*_1_ weighted-imaging; *T*_2_W, *T*_2_ weighted imaging.

Transperineal FLA is mostly performed under general or spinal anesthesia, where patients are placed in a lithotomy position. The laser fibers are usually placed with the help of a transperineal template grid. Transrectal FLA is commonly performed under local anesthesia or under sedation as an outpatient procedure, where patients are placed in prone position. Figure 1 shows the images of a patient that underwent a transrectal FLA procedure. Currently, the Visualase system (Biotex/ Medtronic, Houston, Texas) is mostly used as the laser system, however other systems (*i.e.* Biolitec, CLS) become available for FLA.

The use of FLA appears to be a safe and feasible option for patients with MRI-visible and biopsy-proven localized low- to intermediate-risk prostate cancer who are eligible for both active surveillance (AS) or radical treatment,^14^ but can also be used in a salvage setting.^15^ A recent study by Walser et al demonstrated a freedom of retreatment rate of 83% after a 1 year follow-up in a group of 120 men with low- to intermediate-risk disease that underwent transrectal FLA with no significant changes in quality of life or sexual and urinary function.^16^ Despite recent studies demonstrating large numbers of low- and intermediate-risk disease, the long-term follow-up is still lacking, and therefore patient selection and eligibility criteria need to be carefully evaluated based on which patients will benefit the most.^17–19^

### Cryoablation

Cryoablation is based on the administration of alternating freeze and thaw cycles which will induce cell death and subsequently destruction of the targeted tissue. Tissue injury mainly occurs by cellular dehydration due to the decreased temperature (−40°C) of extracellular water and results in an osmotic gradient followed by coagulative necrosis, thrombosis and tissue ischemia. The process is enhanced by intracellular formation of ice crystals, causing a complete cell disruption.^20^

Cryoablation is performed under general or spinal anesthesia with the patient placed in lithotomy position. Traditionally, the cryoprobes are transperineally inserted using TRUS guidance as it allows real-time feedback on probe positions as well as ice-ball formation. The number of cryoprobes needed depends on the size and shape of the intended ablation zone as well as the type of probe that is used. Nowadays, two prostate cryotherapy systems are used (Visual-ICE^®^ by Galil Medical, Inc and Endocare Cryocare SL^®^ by HealthTronics, Inc.).^21^ Monitoring the ice-ball formation using TRUS is limited due to acoustic shadowing. Consequently, the completed coverage of the ice-ball remains unknown, which may lead to detrimental freezing of the adjacent structures.

**Figure 2.**
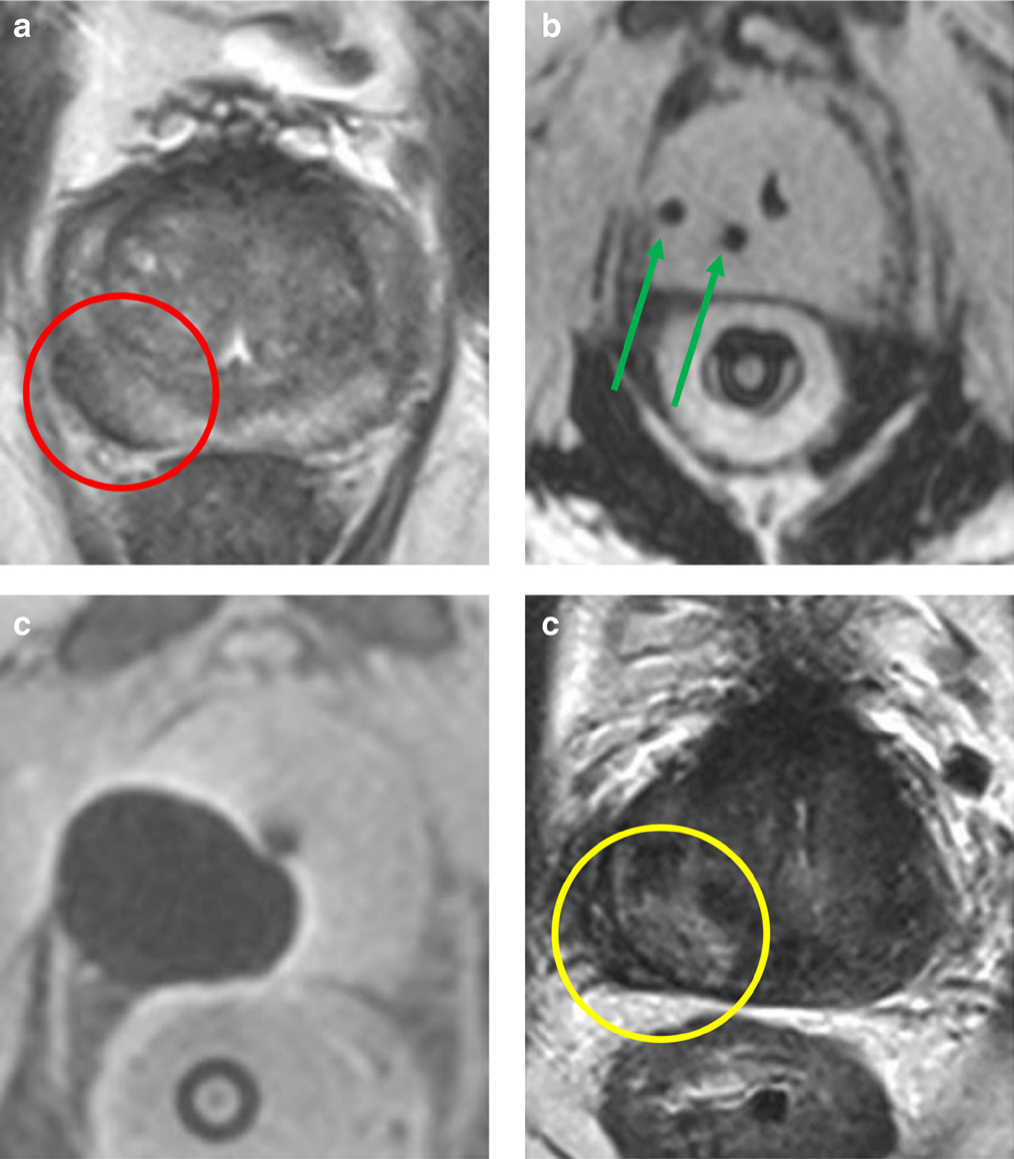
Cryoablation MRI of a 77-year-old male with an initial PSA of 11.4 ng ml^−1^ that underwent EBRT in 2012 as treatment for a *de novo* lesion (Gleason score 4+4=8) at the right peripheral zone. During follow-up, the serum PSA levels increased from 1.1 to 2.7 ng/ml. Multiparametric MRI demonstrated a recurrent lesion at the right peripheral zone (PI-RADS 4). Patient underwent cryotherapy for a recurrent Gleason score 4+5=9 prostate tumor. (a) Axial *T*_2_W imaging demonstrates the prostate tumor in the right peripheral zone (red circle); (b) Intraprocedural axial *T*_1_W imaging with two cryo needles *in situ* (green arrows); (c) Axial *T*_1_W imaging directly after treatment demonstrates the ablation zone; (d) Axial *T*_2_W imaging 1 year after treatment shows the covered area (yellow circle). Serum PSA-levels have decreased to 0.5 ng ml^−1^ and targeted biopsy of the treatment zone showed no residual disease. PI-RADS, Prostate imaging-reporting and data system; PSA, Prostate specific antigen; *T*_1_W, *T*_1_ weighted imaging; *T_2_* W*, T*_2_ weighted imaging.

Therefore, MRI-guidance gained acceptance in the last decade. MR imaging allows extended visualization of the ablation zone due to the possibility of multiplanar imaging and the excellent contrast between ice-ball formation and surrounding tissues.^22^ Next to this, the hyperintense rim around the ice-ball represents with the 0 degree border and can be useful to maintain a safety margin from critical structures during freezing.^23^ Figure 2 demonstrates a patient undergoing MRI-guided cryoablation.

Conventionally, cryoablation is used as whole gland treatment for localized prostate cancer. However, the use of cryoablation as a partial gland or targeted approach is emerging. Mendez et al^24^ found that males with low-risk prostate cancer undergoing focal cryoablation accomplish similar mid-term oncological results with improved erectile function recovery compared to whole gland cryotherapy.

Furthermore, the role of cryoablation as an alternative salvage treatment in patients with local recurrence after radiation therapy is increasing.^25,26^ However, despite the promising results, appropriate patient selection and follow-up after focal cryoablation remaifocns controversial.

### High-intensity focused ultrasound

High-intensity focused ultrasound (HIFU) is a rapidly growing minimal non-invasive partial gland treatment option. Energy from a high-frequency ultrasound transducer (Sonablate^™^; Ablatherm^™^; or FocalOne^™^) is used to heat (>60°C) and destroy the targeted prostatic tissue. The focused ultrasound pulses induce immediate and irreversible tumor necrosis and cause cavitation with sharply delineated margins. A HIFU procedure is performed under local or general anesthesia while using real-time TRUS, MRI or MRI/TRUS-fusion guidance for positioning and monitoring of the transrectally inserted probe. The damage that is caused by using HIFU is limited to the heated tissue, therefore it aims to lower the risk of treatment related side-effects while preserving quality of life and potency.^27^ This also allows secondary treatment using HIFU or additional radical salvage therapy if necessary.^28^ Because the penetration depth of the HIFU pulses is limited, HIFU is considered less suitable for large prostates (>40 cc) or for anterior tumors. Sometimes, a transurethral resection of the prostate (TURP) is performed before HIFU to diminish the prostate volume and to make the prostate eligible for HIFU-treatment.^29^

To date, HIFU is mostly used as focal prostate cancer treatment in both *de novo* prostate cancer as well as a salvage therapy. It appears to be a feasible option with an acceptable survival and oncological outcome in the medium term (5 years) for patients with clinically significant nonmetastatic disease.^30,31^ The oncological outcomes demonstrate an improvement over time, indicating that selection criteria and expertise of the physician are pivotal in the application of focal HIFU.

### Transurethral ultrasound ablation

**Figure 3.**
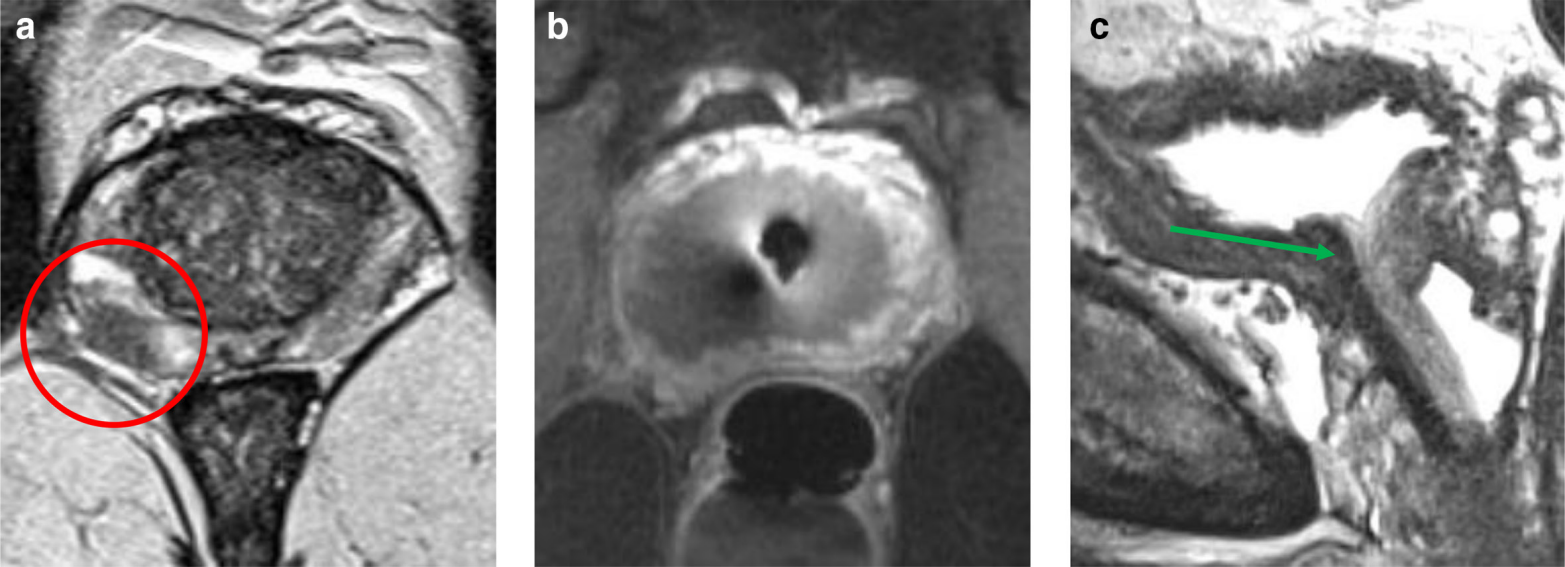
Transurethral ultrasound ablation MRI of a 69-year-old male with an initial PSA of 6.0 ng ml^−1^. Patient underwent MRI-guided whole gland TULSA as treatment for a Gleason score 3+4=7 lesion at the right peripheral zone (PI-RADS 5). (a) Axial *T*_2_W imaging with the prostate tumor (red circle) at the right peripheral zone; (b) Axial *T*_1_W imaging directly after TULSA demonstrates the non-enhancing treatment area with post-treatment edema; (c) Sagittal *T*_2_W imaging 1 year after the TULSA, demonstrating complete removal of the prostate while the urethra has been spared (green arrow). PI-RADS, Prostate imaging-reporting and data system; PSA, Prostate-specific antigen; *T*_1_W, *T*_1_ weighted imaging; *T*_2_W, *T*_2_ weighted imaging; TULSA, Transurethral ultrasound ablation.

MRI-guided transurethral ultrasound ablation (TULSA) is a novel procedure using the TULSA-PRO^®^ technology. It combines MR-based treatment planning and real-time monitoring with thermometry while ablating prostate tissue using transurethral thermal ultrasound.^32–34^ By inducing thermal coagulation of the prostatic tissue, TULSA can deliver accurate and precise whole gland or partial gland ablation. Treatment with TULSA differs from HIFU by delivering a continuous targeted ultrasound beam directly from the urethra, rather than various transrectal focused ultrasound spots. The TULSA-PRO^®^ technology includes a urethral applicator with a linear array of 10 ultrasound transducers that provides a beam of focused energy into the prostatic tissue in order to achieve a temperature of >55°C in the targeted tissue. The urethral applicator and endorectal cooling device facilitate periurethral and rectal preservation by active water cooling.^35^ The TULSA procedure can be performed as outpatient procedure and requires general or spinal anesthesia. Figure 3 displays the MR-imaging of a patient that underwent MRI-guided TULSA. The total procedure from patient positioning to recovery takes almost 4 h with an ablation time of approximately 1 h. Half of the patients can be discharged from the hospital on the day of treatment.^36^

A large prospective multicenter study by Klotz et al^36^ describes whole gland MRI-guided transurethral ultrasound ablation in 115 men with predominately intermediate-risk prostate cancer. While sparing the urethra and apical sphincter, the MRI-guided TULSA demonstrated PSA reduction combined with a low morbidity-rate. Targeted sampling of the treatment area during the 1-year follow-up demonstrates no residual or recurrent tumor in almost 80% of the patients. TULSA also benefits the preservation of the adjacent tissues with a relatively low risk of functional problems. None of the patients had a rectal injury, 96% returned to baseline urinary continence and 75% of potent males maintained or returned to erections sufficient for penetration. Recently, Attinen et al^37^ showed promising early-stage oncological control and low toxicity in patients undergoing TULSA as salvage treatment for local recurrence after primary radiation. 90% of the patients were free of disease in the targeted ablation area one year after treatment.

Despite the promising results, further studies on long-term oncological outcome and treatment-related side-effects are still warranted. Moreover, studies investigating the potential of focal TULSA are still ongoing and the results have not yet been published.

### Photodynamic therapy

Photodynamic therapy (PDT) is a minimally invasive prostate cancer treatment. Its principle is based on three main components: a photosensitizer, light, and tissue oxygen. After local administration in the prostate, a photosensitizing drug is activated by low-power laser light delivered by optical fibers. The optical fibers are placed under TRUS or MRI/TRUS guidance using a needle and a perineal template as guidance. Transfer of energy from the activated photosensitizing drug to biological substrates or molecular oxygen, generates reactive oxygen species which induces cell death by apoptosis or necrosis.^38^ Consequently, PDT has shown to initiate selective cytotoxicity towards malignant cells and subsequently leads to malignant cell death.^39^ The commonly used photosensitizers differ in form of administration (intravenous, oral or topical), wavelength required for activation (405–763 nm) and targeted tissue (tissue-based or vascular acting).^40^ However, photosensitivity and phototoxicity are important factors associated with PDT and need to be considered as main limitations. The intracellular accumulation of the photosensitizing drug inside of cells might lead to increased susceptibility to prolonged phototoxicity, resulting in an extreme sensitivity to ultraviolet (UV)-rays from the sun and other light sources.^40^

Photodynamic therapy has the potential to be very targeted and provides a single session treatment, nevertheless it can also be used in primary (whole gland) or salvage settings.^41^ Compared to active surveillance, PDT demonstrated to be a safe and an effective tissue-preserving approach for low-risk localized prostate cancer. In these cases, PDT may even defer or avoid radical therapy.^42,43^ Nowadays, studies are mainly focusing on applying PDT as treatment of localized advanced prostate cancer and isolated metastases.^44,45^ Treatment of advanced prostate cancer using PDT has the potential to establish annihilation of the malignant tissue and reduce damage to adjacent structures. The oncological efficacy of PDT on androgen-refractory prostate cancer is especially important for prostate tumors or patients resistant to hormonal therapy.^46^ The use of PDT also appears suitable for organ confined recurrent prostate cancer after radiation therapy, destroying essentially all glandular tissue within the prostate under precise light dosimetry with only a few complications.^47^ However, the chronic and cumulative toxicity that is associated with radiation therapy does not occur during PDT. More importantly, PDT can be applied to already irradiated prostatic tissue.

### Radiofrequency ablation

Radiofrequency ablation (RFA) or radiofrequency interstitial tumor ablation (RITA) is an innovative targeted treatment that precisely delivers low-dose radiofrequency waves directly to cancerous tissue. The targeted tissue is irreversibly destroyed by the established coagulative necrosis and atrophy.^48^ Prostate cancer treatment using RFA can be an effective and safe option for patients with clinically localized prostate cancer as well as patients with non-metastatic recurrent disease.^49^ It is used as primary or salvage therapy for cancers that are not eligible for surgical removal and a common treatment option in case of therapy-resistant tumors.

Traditionally, RFA is performed under general or spinal anesthesia, where patients are placed in a gynecological position for a transperineally approach. A transurethral catheter is needed to improve ultrasonographic visualization. The needles are placed under TRUS or MRI/TRUS fusion guidance while using a transperineal template grid. A generator (RITA Medical Systems Inc, California) provides monopolar or bipolar radiofrequency energy up to 50 W at a frequency of 480 kHz.^50^ Recently, Orczyk et al^51^ reported on focal MRI/TRUS fusion-guided bipolar radiofrequency ablation for clinically significant prostate cancer visible on mpMRI in a group of 20 patients. This bipolar “Encage” system (Trod Medical, Bradenton, FL) uses a novel asymmetrical coil which creates a precise uniform zone of coagulative necrosis. 6 months after treatment, 80% (*n* = 16) was free of clinically significant prostate cancer on targeted transperineal biopsy. Absence of erectile dysfunction was seen in 91.7% of the patients with no erectile dysfunction at baseline. The return to baseline mainly occurred during the first 6 weeks after treatment. Scores for intercourse satisfaction, sexual desire, overall sexual satisfaction and orgasmic function did not demonstrate any changes. In 89% (16/18) of the males with no urinary incontinence at baseline the absence of urinary incontinence remained after treatment. Furthermore, they reported no change in lower urinary tract symptoms, bowel habit, general health and prostate related quality of life. The use of focal RFA is a feasible and safe treatment option for patients with localized prostate cancer. However, further research with extended follow-up is needed to evaluate oncological efficacy and treatment related side-effects.^52^

### Irreversible electroporation

Irreversible electroporation (IRE) is a relatively novel non-thermal ablation technique where micro- to millisecond electrical pulses travel between transperineally inserted electrodes.^53^ The electrical pulses produce irreversible cell membrane permeabilization which causes apoptosis of the cells. Therefore, it only affects the cell membrane while preserving the surrounding tissues and the extracellular matrix.

IRE procedure is performed under general anesthesia and deep muscle paralysis using TRUS or MRI/TRUS–fusion technique for electrode placement. Patients are positioned in lithotomy position and a transurethral urinary catheter needs to be placed. The positioning and guidance of the electrodes requires a transperineal template grid. Most IRE procedures are performed by using a widely available IRE generator with planning software (NanoKnife; Angiodynamics, New York, NY). The average procedure time using IRE is approximately 1 h.^54^

Multiple Phase I–II studies have shown that IRE is a safe and feasible focal treatment option with a low morbidity rate in both primary and salvage diseases.^54–56^ Blazevski et al^57^ demonstrated a disease-free survival rate of 97.3% in 123 patients with localized clinically significant prostate cancer. They also defined that the ideal patient should have a biopsy proven intermediate-risk localized prostate cancer with a unifocal lesion on mpMRI. A study by Collettini et al^58^ showed a similar cancer-free survival rate of 82% 6 months after IRE. 1-year follow-up data demonstrated a significant reduction in the serum PSA-levels, a stable urogenital function, and a leak- and pad-free continence rate of 96.3%. Remarkably, Scheltema et al^59^ experienced more early oncological failure after IRE in a study that compared IRE (*n* = 50) to robot-assisted radical prostatectomy (RARP) (*n* = 50). However, they demonstrated superior preservation of pad-free continence (UC) and erections sufficient for intercourse (ESI) in the group that underwent IRE. 12 months after treatment, urinary symptoms had been reduced for both groups, although the patients undergoing IRE initially had more urinary symptoms directly after treatment. Additionally, males with a poor baseline functioning are more likely to develop erectile dysfunction after IRE. Despite the promising oncological outcomes, long-term follow-up data of oncological efficacy and treatment-related complications and side-effects is currently not available.

### Brachytherapy

**Figure 4.**
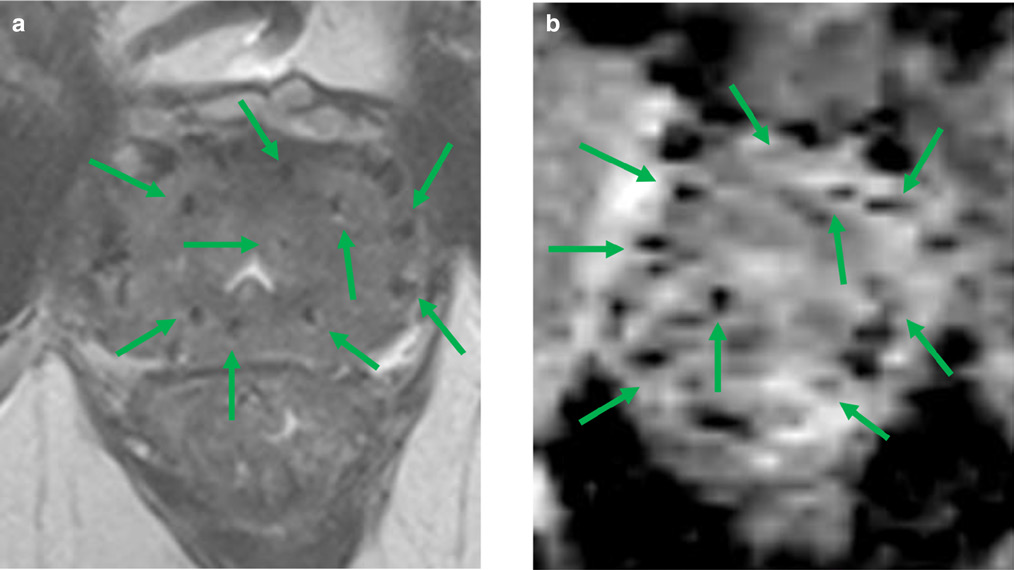
Imaging after brachytherapy MRI of a 68-year-old male with an initial PSA of 7.4 ng ml^−1^ after brachytherapy in 2012 as treatment for a Gleason score 3+4=7 lesion at the left peripheral zone. (a) Axial *T*_2_W imaging demonstrates the characteristic ellipse shaped brachy seeds (green arrows); (b) Axial ADC map. ADC, Apparent diffusion coefficient; PSA, Prostate specific antigen; *T*_2_W, *T*_2_ weighted imaging.

The principle of brachytherapy (or internal radiation therapy) is based on radioactive seeds that are implanted within the prostatic tissue under image guidance. Brachytherapy can be distinguished by high-dose rate therapy (HDR) or low-dose rate therapy (LDR). High-dose rate brachytherapy is delivered in a brief treatment session where radioactive pellets are temporarily inserted. This results in accurate dosimetry as it allows modulation of the source dwell time and implant position. Alternatively, LDR brachytherapy allows definitive implantation of tiny radioactive titanium seeds and is more commonly used as it is most suitable for low-risk prostate cancer in low volume prostate tissue. Figure 4 demonstrates the MR-imaging of a patient that underwent brachytherapy.

Brachytherapy can be used as whole gland or partial gland treatment of a prostate tumor with fewer side-effects than, *i.e.* EBRT due to a specific distribution of high dose radiation.^60^ However, brachytherapy may also be combined with EBRT in order to improve prostate cancer treatment.^61^ Partial gland therapy using brachytherapy is mainly performed transperineally under either MRI or MRI/TRUS-fusion guidance while using a biopsy template.^62,63^ During treatment under local, spinal or general anesthesia, the patient is placed in lithotomy position and a transurethral foley catheter is inserted. In case of cognitive or rigid fusion systems, the mobility and deformation of the prostate influence the precise targeted area, Therefore, the implantation of brachy seeds using elastic fusion registration is evaluated. This ultra-focal HDR brachytherapy, uses a single ancillary ultrasound visible marker that is transrectally inserted within or close to the targeted region prior to routine brachytherapy.^62^ A recent study by Graff et al^62^ researched precision delivery of therapeutic radiation doses to small tumor lesions on mpMRI.

Mid-term results of treatment with MRI-guided brachytherapy indicate that it is feasible to treat tumors, while limiting toxicity and preserving quality of life.^63^ Most importantly, targeted lesions in the base of the prostate are more likely correlated with urinary symptoms compared to lesions located in the apex during early follow-up after focal brachytherapy.^64^ Focal brachytherapy is an emerging prostate cancer treatment which still requires further evaluation to conquer imprecision in the definition of the target (location, shape, and volume) and to ensure collaboration between the imaging modalities required for targeted treatment.

### Radiation therapy

Radiation therapy (RT) is generally used as radical treatment for localized prostate cancer in males not eligible for surgery.^65^ External beam radiotherapy (EBRT) and its hypofractionation equivalent, stereotactic body radiation therapy (SBRT), are currently widely used in approximately one-third of patients with localized (cT1c-T3N0M0) prostate cancer.^66^ However, safe delivery of the intended fraction for tumor tissue in the abdominal and pelvic region is limited due to inter- and intrafractional organ movement. The use of MR-guided radiotherapy (MRgRT) as stereotactic therapy allows for greater accuracy of fraction delivery using better soft-tissue contrast, real-time MR imaging for direct tracking and daily online adaptive planning software.^67^ This will improve cancer outcome while reducing the risk of treatment-related toxicity and additional radiation exposure.^68^ To date, two commercially available systems are used: Elekta Unity (Elekta AB, Stockholm, Sweden) using 1.5 Tesla, and Viewray MRIdian MR Linacs (Viewray Inc, Oakwood, OH) using a 0.35 Tesla MRI.^69^

Magnetic resonance-guided radiotherapy has shown promising results as a safe and tolerable prostate cancer treatment.^70,71^ The average MRgRT dose delivery takes approximately 45 min and has dosimetry benefits over other forms of radiotherapy.^72^ A Phase-II study (*n* = 101) reported a low incidence of early gastrointestinal and genitourinary toxicity using both clinician- and patient-reported outcome measurements in patients with localized prostate cancer undergoing MRgRT.^71^ Follow-up data of one study provide extended toxicity information with symptoms no longer present 12 months after treatment.^72^ However, studies in a large cohort of patients and long-term follow-up regarding oncologic outcomes are lacking.

## Conclusion

This review provides an overview of image-guided prostate cancer treatment options while using MR-, TRUS- or MRI/TRUS fusion imaging. Minimal invasive, image-guided prostate interventions may be considered as a viable partial gland ablation option compared with the current, standard treatment options in patients with organ confined low- to intermediate-risk prostate cancer (recurrence). Correct utilization of a treatment in patients that will benefit the most while ensuring a safe and responsible application by physicians has become the main challenge. Majority of the studies focusing on minimally invasive prostate cancer treatments only report early stages of research and high-level evidence is still lacking. Despite the promising results and emerging evidence, definite proof of oncological efficacy compared to radical treatment using randomized controlled trials is required.

## References

[1] Mottet N, Bellmunt J, Bolla M, Briers E, Cumberbatch MG, De Santis M, et al . EAU-ESTRO-SIOG guidelines on prostate cancer. Part 1: screening, diagnosis, and local treatment with curative intent. Eur Urol 2017; 71: 618–29. doi: 10.1016/j.eururo.2016.08.003 27568654

[2] Hull GW, Rabbani F, Abbas F, Wheeler TM, Kattan MW, Scardino PT . Cancer control with radical prostatectomy alone in 1,000 consecutive patients. J Urol 2002; 167(2 Pt 1): 528–34. doi: 10.1016/S0022-5347(01)69079-7 11792912

[3] Stephenson AJ, Scardino PT, Eastham JA, Bianco FJ, Dotan ZA, Fearn PA, et al . Preoperative nomogram predicting the 10-year probability of prostate cancer recurrence after radical prostatectomy. J Natl Cancer Inst 2006; 98: 715–7. doi: 10.1093/jnci/djj190 16705126 PMC2242430

[4] Sanda MG, Dunn RL, Michalski J, Sandler HM, Northouse L, Hembroff L, et al . Quality of life and satisfaction with outcome among prostate-cancer survivors. N Engl J Med 2008; 358: 1250–61. doi: 10.1056/NEJMoa074311 18354103

[5] Resnick MJ, Koyama T, Fan K-H, Albertsen PC, Goodman M, Hamilton AS, et al . Long-Term functional outcomes after treatment for localized prostate cancer. N Engl J Med 2013; 368: 436–45. doi: 10.1056/NEJMoa1209978 23363497 PMC3742365

[6] Bozzini G, Colin P, Nevoux P, Villers A, Mordon S, Betrouni N . Focal therapy of prostate cancer: energies and procedures. Urol Oncol 2013; 31: 155–67. doi: 10.1016/j.urolonc.2012.05.011 22795500

[7] Eggener S, Salomon G, Scardino PT, De la Rosette J, Polascik TJ, Brewster S . Focal therapy for prostate cancer: possibilities and limitations. Eur Urol 2010; 58: 57–64. doi: 10.1016/j.eururo.2010.03.034 20378241

[8] Ahmed HU, Dickinson L, Charman S, Weir S, McCartan N, Hindley RG, et al . Focal ablation targeted to the index lesion in multifocal localised prostate cancer: a prospective development study. Eur Urol 2015; 68: 927–36. doi: 10.1016/j.eururo.2015.01.030 25682339

[9] Rosenkrantz AB, Verma S, Choyke P, Eberhardt SC, Eggener SE, Gaitonde K, et al . Prostate magnetic resonance imaging and magnetic resonance imaging targeted biopsy in patients with a prior negative biopsy: a consensus statement by AUA and SAR. J Urol 2016; 196: 1613–8. doi: 10.1016/j.juro.2016.06.079 27320841 PMC6364689

[10] European Association U .European association of urology guidelines. 2018 edition. Arnhem, The Netherlands: European Association of Urology Guidelines Office; 2018.

[11] Patel P, Mathew MS, Trilisky I, Oto A . Multiparametric MR imaging of the prostate after treatment of prostate cancer. Radiographics 2018; 38: 437–49. doi: 10.1148/rg.2018170147 29373089

[12] Mertan FV, Greer MD, Borofsky S, Kabakus IM, Merino MJ, Wood BJ, et al . Multiparametric magnetic resonance imaging of recurrent prostate cancer. Top Magn Reson Imaging 2016; 25: 139–47. doi: 10.1097/RMR.0000000000000088 27187164 PMC5462597

[13] Bomers JGR, Sedelaar JPM, Barentsz JO, Fütterer JJ . Mri-Guided interventions for the treatment of prostate cancer. AJR Am J Roentgenol 2012; 199: 714–20. doi: 10.2214/AJR.12.8725 22997360

[14] Eggener SE, Yousuf A, Watson S, Wang S, Oto A . Phase II evaluation of magnetic resonance imaging guided focal laser ablation of prostate cancer. J Urol 2016; 196: 1670–5. doi: 10.1016/j.juro.2016.07.074 27449263

[15] Woodrum DA, Kawashima A, Gorny KR, Mynderse LA . Prostate cancer: state of the art imaging and focal treatment. Clin Radiol 2017; 72: 665–79. doi: 10.1016/j.crad.2017.02.010 28385253

[16] Walser E, Nance A, Ynalvez L, Yong S, Aoughsten JS, Eyzaguirre EJ, et al . Focal laser ablation of prostate cancer: results in 120 patients with low- to intermediate-risk disease. J Vasc Interv Radiol 2019; 30: 401–9. doi: 10.1016/j.jvir.2018.09.016 30819483 PMC8503805

[17] van Luijtelaar A, Greenwood BM, Ahmed HU, Barqawi AB, Barret E, Bomers JGR, et al . Focal laser ablation as clinical treatment of prostate cancer: report from a Delphi consensus project. World J Urol 2019; 37: 2147–53. doi: 10.1007/s00345-019-02636-7 30671638 PMC6763411

[18] Valerio M, Ahmed HU, Emberton M, Lawrentschuk N, Lazzeri M, Montironi R, et al . The role of focal therapy in the management of localised prostate cancer: a systematic review. Eur Urol 2014; 66: 732–51. doi: 10.1016/j.eururo.2013.05.048 23769825 PMC4179888

[19] Mehralivand S, George AK, Hoang AN, Rais-Bahrami S, Rastinehad AR, Lebastchi AH, et al . MRI-Guided focal laser ablation of prostate cancer: a prospective single-arm, single-center trial with 3 years of follow-up. Diagn Interv Radiol 2021; 27: 394–400. doi: 10.5152/dir.2021.20095 34003127 PMC8136525

[20] Finley DS, Pouliot F, Miller DC, Belldegrun AS . Primary and salvage cryotherapy for prostate cancer. Urol Clin North Am 2010; 37: 67–82. doi: 10.1016/j.ucl.2009.11.007 20152521

[21] Lau B, Shah TT, Valerio M, Hamid S, Ahmed HU, Arya M . Technological aspects of delivering cryotherapy for prostate cancer. Expert Rev Med Devices 2015; 12: 183–90. doi: 10.1586/17434440.2015.990377 25569713

[22] Woodrum DA, Kawashima A, Karnes RJ, Davis BJ, Frank I, Engen DE, et al . Magnetic resonance imaging-guided cryoablation of recurrent prostate cancer after radical prostatectomy: initial single institution experience. Urology 2013; 82: 870–5. doi: 10.1016/j.urology.2013.06.011 23910089

[23] Overduin CG, Bomers JGR, Jenniskens SFM, Hoes MF, Ten Haken B, de Lange F, et al . T1-Weighted Mr image contrast around a cryoablation iceball: a phantom study and initial comparison with in vivo findings. Med Phys 2014; 41: 112301. doi: 10.1118/1.4896824 25370657

[24] Mendez MH, Passoni NM, Pow-Sang J, Jones JS, Polascik TJ . Comparison of outcomes between preoperatively potent men treated with focal versus whole gland cryotherapy in a matched population. J Endourol 2015; 29: 1193–8. doi: 10.1089/end.2014.0881 26058496

[25] Bomers JGR, Yakar D, Overduin CG, Sedelaar JPM, Vergunst H, Barentsz JO, et al . Mr imaging-guided focal cryoablation in patients with recurrent prostate cancer. Radiology 2013; 268: 451–60. doi: 10.1148/radiol.13121291 23525206

[26] de Castro Abreu AL, Bahn D, Leslie S, Shoji S, Silverman P, Desai MM, et al . Salvage focal and salvage total cryoablation for locally recurrent prostate cancer after primary radiation therapy. BJU Int 2013; 112: 298–307. doi: 10.1111/bju.12151 23826840

[27] Blana A, Murat FJ, Walter B, Thuroff S, Wieland WF, Chaussy C, et al . First analysis of the long-term results with transrectal HIFU in patients with localised prostate cancer. Eur Urol 2008; 53: 1194–203. doi: 10.1016/j.eururo.2007.10.062 17997026

[28] Stabile A, Orczyk C, Hosking-Jervis F, Giganti F, Arya M, Hindley RG, et al . Medium-Term oncological outcomes in a large cohort of men treated with either focal or hemi-ablation using high-intensity focused ultrasonography for primary localized prostate cancer. BJU Int 2019; 124: 431–40. doi: 10.1111/bju.14710 30753756

[29] Garcia-Barreras S, Sanchez-Salas R, Sivaraman A, Barret E, Secin F, Nunes-Silva I, et al . Comparative analysis of partial gland ablation and radical prostatectomy to treat low and intermediate risk prostate cancer: oncologic and functional outcomes. J Urol 2018; 199: 140–6. doi: 10.1016/j.juro.2017.08.076 28823768

[30] Guillaumier S, Peters M, Arya M, Afzal N, Charman S, Dudderidge T, et al . A multicentre study of 5-year outcomes following focal therapy in treating clinically significant nonmetastatic prostate cancer. Eur Urol 2018; 74: 422–9. doi: 10.1016/j.eururo.2018.06.006 29960750 PMC6156573

[31] Tourinho-Barbosa RR, Sanchez-Salas R, Claros OR, Collura-Merlier S, Bakavicius A, Carneiro A, et al . Focal therapy for localized prostate cancer with either high intensity focused ultrasound or cryoablation: a single institution experience. J Urol 2020; 203: 320–30. doi: 10.1097/JU.0000000000000506 31437121

[32] Burtnyk M, Hill T, Cadieux-Pitre H, Welch I . Magnetic resonance image guided transurethral ultrasound prostate ablation: a preclinical safety and feasibility study with 28-day followup. J Urol 2015; 193: 1669–75. doi: 10.1016/j.juro.2014.11.089 25464003

[33] Chopra R, Colquhoun A, Burtnyk M, N'djin WA, Kobelevskiy I, Boyes A, et al . MR imaging-controlled transurethral ultrasound therapy for conformal treatment of prostate tissue: initial feasibility in humans. Radiology 2012; 265: 303–13. doi: 10.1148/radiol.12112263 22929332

[34] Siddiqui K, Chopra R, Vedula S, Sugar L, Haider M, Boyes A, et al . MRI-Guided transurethral ultrasound therapy of the prostate gland using real-time thermal mapping: initial studies. Urology 2010; 76: 1506–11. doi: 10.1016/j.urology.2010.04.046 20709381

[35] Chin JL, Billia M, Relle J, Roethke MC, Popeneciu IV, Kuru TH, et al . Magnetic resonance imaging-guided transurethral ultrasound ablation of prostate tissue in patients with localized prostate cancer: a prospective phase 1 clinical trial. Eur Urol 2016; 70: 447–55. doi: 10.1016/j.eururo.2015.12.029 26777228

[36] Klotz L, Pavlovich CP, Chin J, Hatiboglu G, Koch M, Penson D, et al . Magnetic resonance imaging-guided transurethral ultrasound ablation of prostate cancer. J Urol 2021; 205: 769–79. doi: 10.1097/JU.0000000000001362 33021440

[37] Anttinen M, Mäkelä P, Viitala A, Nurminen P, Suomi V, Sainio T, et al . Salvage magnetic resonance imaging-guided transurethral ultrasound ablation for localized radiorecurrent prostate cancer: 12-month functional and oncological results. Eur Urol Open Sci 2020; 22: 79–87. doi: 10.1016/j.euros.2020.10.007 34337481 PMC8317885

[38] Yoon I, Li JZ, Shim YK . Advance in photosensitizers and light delivery for photodynamic therapy. Clin Endosc 2013; 46: 7–23. doi: 10.5946/ce.2013.46.1.7 23423543 PMC3572355

[39] Agostinis P, Berg K, Cengel KA, Foster TH, Girotti AW, Gollnick SO, et al . Photodynamic therapy of cancer: an update. CA Cancer J Clin 2011; 61: 250–81. doi: 10.3322/caac.20114 21617154 PMC3209659

[40] Gheewala T, Skwor T, Munirathinam G . Photosensitizers in prostate cancer therapy. Oncotarget 2017; 8: 30524–38. doi: 10.18632/oncotarget.15496 28430624 PMC5444762

[41] Ahmed HU, Moore C, Emberton M . Minimally-invasive technologies in uro-oncology: the role of cryotherapy, HIFU and photodynamic therapy in whole gland and focal therapy of localised prostate cancer. Surg Oncol 2009; 18: 219–32. doi: 10.1016/j.suronc.2009.02.002 19268572

[42] Azzouzi A-R, Vincendeau S, Barret E, Cicco A, Kleinclauss F, van der Poel HG, et al . Padeliporfin vascular-targeted photodynamic therapy versus active surveillance in men with low-risk prostate cancer (CLIN1001 PCM301): an open-label, phase 3, randomised controlled trial. Lancet Oncol 2017; 18: 181–91. doi: 10.1016/S1470-2045(16)30661-1 28007457

[43] Gill IS, Azzouzi A-R, Emberton M, Coleman JA, Coeytaux E, Scherz A, et al . Randomized trial of partial gland ablation with vascular targeted phototherapy versus active surveillance for low risk prostate cancer: extended followup and analyses of effectiveness. J Urol 2018; 200: 786–93. doi: 10.1016/j.juro.2018.05.121 29864437 PMC6786489

[44] Koudinova NV, Pinthus JH, Brandis A, Brenner O, Bendel P, Ramon J, et al . Photodynamic therapy with Pd-Bacteriopheophorbide (Tookad): successful in vivo treatment of human prostatic small cell carcinoma xenografts. Int J Cancer 2003; 104: 782–9. doi: 10.1002/ijc.11002 12640688

[45] Momma T, Hamblin MR, Wu HC, Hasan T . Photodynamic therapy of orthotopic prostate cancer with benzoporphyrin derivative: local control and distant metastasis. Cancer Res 1998; 58: 5425–31. 9850075

[46] Xu DD, Lam HM, Hoeven R, Xu CB, Leung AWN, Cho WCS, DD X, , WCS C . Photodynamic therapy induced cell death of hormone insensitive prostate cancer PC-3 cells with autophagic characteristics. Photodiagnosis Photodyn Ther 2013; 10: 278–87. doi: 10.1016/j.pdpdt.2013.01.002 23993854

[47] Nathan TR, Whitelaw DE, Chang SC, Lees WR, Ripley PM, Payne H, et al . Photodynamic therapy for prostate cancer recurrence after radiotherapy: a phase I study. J Urol 2002; 168(4, Part 1): 1427–32. doi: 10.1016/S0022-5347(05)64466-7 12352410

[48] Curley SA, Izzo F . Radiofrequency ablation of hepatocellular carcinoma. Minerva Chir 2002; 57: 165–76. 11941292

[49] Shariat SF, Raptidis G, Masatoschi M, Bergamaschi F, Slawin KM . Pilot study of radiofrequency interstitial tumor ablation (RITA) for the treatment of radio-recurrent prostate cancer. Prostate 2005; 65: 260–7. doi: 10.1002/pros.20242 16015591

[50] Zlotta AR, Djavan B, Matos C, Noel JC, Peny MO, Silverman DE, et al . Percutaneous transperineal radiofrequency ablation of prostate tumour: safety, feasibility and pathological effects on human prostate cancer. Br J Urol 1998; 81: 265–75. doi: 10.1046/j.1464-410X.1998.00504.x 9488071

[51] Orczyk C, Barratt D, Brew-Graves C, Peng Hu Y, Freeman A, McCartan N, et al . Prostate radiofrequency focal ablation (ProRAFT) trial: a prospective development study evaluating a bipolar radiofrequency device to treat prostate cancer. J Urol 2021; 205: 1090–9. doi: 10.1097/JU.0000000000001567 33315505

[52] Aydin AM, Gage K, Dhillon J, Cheriyan SK, Poch MA, Manley BJ, et al . Focal bipolar radiofrequency ablation for localized prostate cancer: safety and feasibility. Int J Urol 2020; 27: 882–9. doi: 10.1111/iju.14321 32767444

[53] Rubinsky B, Onik G, Mikus P . Irreversible electroporation: a new ablation modality--clinical implications. Technol Cancer Res Treat 2007; 6: 37–48. doi: 10.1177/153303460700600106 17241099

[54] Blazevski A, Scheltema MJ, Amin A, Thompson JE, Lawrentschuk N, Stricker PD . Irreversible electroporation (IRE): a narrative review of the development of IRE from the laboratory to a prostate cancer treatment. BJU Int 2020; 125: 369–78. doi: 10.1111/bju.14951 31725935

[55] Valerio M, Dickinson L, Ali A, Ramachadran N, Donaldson I, Mccartan N, et al . Nanoknife electroporation ablation trial: a prospective development study investigating focal irreversible electroporation for localized prostate cancer. J Urol 2017; 197(3 Pt 1): 647–54. doi: 10.1016/j.juro.2016.09.091 27697580

[56] Scheltema MJ, van den Bos W, Siriwardana AR, Kalsbeek AMF, Thompson JE, Ting F, et al . Feasibility and safety of focal irreversible electroporation as salvage treatment for localized radio-recurrent prostate cancer. BJU Int 2017; 120 Suppl 3(Suppl 3): 51–8. doi: 10.1111/bju.13991 28834167

[57] Blazevski A, Scheltema MJ, Yuen B, Masand N, Nguyen TV, Delprado W, et al . Oncological and quality-of-life outcomes following focal irreversible electroporation as primary treatment for localised prostate cancer: a Biopsy-monitored prospective cohort. Eur Urol Oncol 2020; 3: 283–90. doi: 10.1016/j.euo.2019.04.008 31103721

[58] Collettini F, Enders J, Stephan C, Fischer T, Baur ADJ, Penzkofer T, et al . Image-Guided irreversible electroporation of localized prostate cancer: functional and oncologic outcomes. Radiology 2019; 292: 250–7. doi: 10.1148/radiol.2019181987 31161973

[59] Scheltema MJ, Chang JI, Böhm M, van den Bos W, Blazevski A, Gielchinsky I, et al . Pair-matched patient-reported quality of life and early oncological control following focal irreversible electroporation versus robot-assisted radical prostatectomy. World J Urol 2018; 36: 1383–9. doi: 10.1007/s00345-018-2281-z 29594551 PMC6105143

[60] Peach MS, Trifiletti DM, Libby B . Systematic review of focal prostate brachytherapy and the future implementation of image-guided prostate HDR brachytherapy using MR-Ultrasound fusion. Prostate Cancer 2016; 2016: 1–13. doi: 10.1155/2016/4754031 27293899 PMC4884850

[61] Sylvester JE, Blasko JC, Grimm PD, Meier R, Malmgren JA . Ten-Year biochemical relapse-free survival after external beam radiation and brachytherapy for localized prostate cancer: the Seattle experience. Int J Radiat Oncol Biol Phys 2003; 57: 944–52. doi: 10.1016/S0360-3016(03)00739-9 14575824

[62] Graff P, Portalez D, Lusque A, Brun T, Aziza R, Khalifa J, et al . Ideal 2A phase II study of Ultrafocal brachytherapy for low- and intermediate-risk prostate cancer. Int J Radiat Oncol Biol Phys 2018; 102: 903–11. doi: 10.1016/j.ijrobp.2018.01.066 29510957

[63] Peters M, van Son MJ, Moerland MA, Kerkmeijer LGW, Eppinga WSC, Meijer RP, et al . MRI-Guided Ultrafocal HDR brachytherapy for localized prostate cancer: median 4-year results of a feasibility study. Int J Radiat Oncol Biol Phys 2019; 104: 1045–53. doi: 10.1016/j.ijrobp.2019.03.032 30926575

[64] Srougi V, Barret E, Nunes-Silva I, Baghdadi M, Garcia-Barreras S, Pierrat N, et al . Focal brachytherapy for localized prostate cancer: urinary toxicity depends on tumor location. Brachytherapy 2017; 16: 988–92. doi: 10.1016/j.brachy.2017.05.009 28648486

[65] Cooperberg MR, Broering JM, Carroll PR . Time trends and local variation in primary treatment of localized prostate cancer. J Clin Oncol 2010; 28: 1117–23. doi: 10.1200/JCO.2009.26.0133 20124165 PMC2834465

[66] Proust-Lima C, Taylor JMG, Sécher S, Sandler H, Kestin L, Pickles T, et al . Confirmation of a low α/β ratio for prostate cancer treated by external beam radiation therapy alone using a post-treatment Repeated-Measures model for PSA dynamics. Int J Radiat Oncol Biol Phys 2011; 79: 195–201. doi: 10.1016/j.ijrobp.2009.10.008 20381268 PMC4122313

[67] Pathmanathan AU, Schmidt MA, Brand DH, Kousi E, van As NJ, Tree AC . Improving fiducial and prostate capsule visualization for radiotherapy planning using MRI. J Appl Clin Med Phys 2019; 20: 27–36. doi: 10.1002/acm2.12529 30756456 PMC6414142

[68] Gill S, Thomas J, Fox C, Kron T, Rolfo A, Leahy M, et al . Acute toxicity in prostate cancer patients treated with and without image-guided radiotherapy. Radiat Oncol 2011; 6: 145. doi: 10.1186/1748-717X-6-145 22035354 PMC3217047

[69] Ménard C, van der Heide U . Introduction: systems for magnetic resonance image guided radiation therapy. Semin Radiat Oncol 2014; 24: 192. doi: 10.1016/j.semradonc.2014.02.010 24931090

[70] Alongi F, Rigo M, Figlia V, Cuccia F, Giaj-Levra N, Nicosia L, et al . 1.5 T MR-guided and daily adapted SBRT for prostate cancer: feasibility, preliminary clinical tolerability, quality of life and patient-reported outcomes during treatment. Radiat Oncol 2020; 15: 69. doi: 10.1186/s13014-020-01510-w 32248826 PMC7092497

[71] Bruynzeel AME, Tetar SU, Oei SS, Senan S, Haasbeek CJA, Spoelstra FOB, et al . A prospective single-arm phase 2 study of stereotactic magnetic resonance guided adaptive radiation therapy for prostate cancer: early toxicity results. Int J Radiat Oncol Biol Phys 2019; 105: 1086–94. doi: 10.1016/j.ijrobp.2019.08.007 31419510

[72] Tetar SU, Bruynzeel AME, Lagerwaard FJ, Slotman BJ, Bohoudi O, Palacios MA . Clinical implementation of magnetic resonance imaging guided adaptive radiotherapy for localized prostate cancer. Phys Imaging Radiat Oncol 2019; 9: 69–76. doi: 10.1016/j.phro.2019.02.002 33458428 PMC7807673

